# Supplementary material for: Patterns of Intron Gain and Loss in Fungi
Source: PLoS Biol. 2004 Nov 30;2(12):e422. doi: 10.1371/journal.pbio.0020422 (PMC532390; doi:10.1371/journal.pbio.0020422)
Supplement: Table S1 — Also available at http://genes.mit.edu/NielsenEtAl/. (4.3 MB ZIP). [file pbio.0020422.st001.zip › NielsenEtAl/html/1181.html]

AN4583.1.NCU03853.1.MG08104.1.FG10352.1


```
 CLUSTAL W (1.82) Multiple Sequence Alignments - Introns Inserted


Sequence 1: NCU03853.1	375 aa
Sequence 2: MG08104.1	376 aa
Sequence 3: FG10352.1	372 aa
Sequence 4: AN4583.1	372 aa
Alignment Length: 381 aa
Number Identitical Residues: 194 aa
Alignment Score (without introns) 9052


MG08104.1 	MSADETNKPAR~PRVFFDISIDDKPAGRIVFEL~FSDV~VPKTAENFRALCTGEKGIGKS
NCU03853.1	MSSTDDVKQAR~SRVFFDITIGGKAAGRIVFEL~YNDI~VPKTAENFRALCTGEKGVGKL
FG10352.1 	-MAAEGDSPAR~PRVYFDISIGGKSVGRITMEL~YADL~VPKTADNFRSLCTGEKGIGKS
AN4583.1  	-----MAESKR1PRVFFDIQIGQQQTGRIAFEL0FNDV1VPKTAENFRALCTGEKGMGKQ
          	       .  * .**:*** *. : .***.:** : *: *****:***:*******:** 

MG08104.1 	GKPLHYKG~SSFHRVIKQFMIQGGDFTAGNGTGGESIYGEKFDDENFELIHDRPFLLSMA
NCU03853.1	GKPLHYKG~STFHRVIKQFMIQGGDFTAGNGTGGESIYGAKFEDENFQLKHDRPFLLSMA
FG10352.1 	GKPLHYKG~SVFHRVIKQFMIQGGDFTAGDGTGGESIYGNKFDDEAFPIKHEKPFLLSMA
AN4583.1  	GKPLHFKG1SIFHRVIKQFMIQGGDFTAFNGTGGESIYGEKFPDENFELKHDRPFLLSMA
          	*****:** * ***************** :********* ** ** * : *::*******

MG08104.1 	NAGPG1TNGSQFFVTTVPTPHLDKKHVVFGQVLSGKSVVRQVENLTTHAGDKPVKSATIA
NCU03853.1	NAGPG1TNGSQFFVTTVPTPHLDGKHVVFGEVLSGKSVVRQIENLKTQ-GDKPTKDAVIA
FG10352.1 	NAGPN1TNGSQFFITTVPTPHLDGKHVVFGEVLNGKSVVRQIENLTTQSGDRPAKEALIV
AN4583.1  	NSGPG~TNGSQFFITTVPTPHLDGKHVVFGEVINGKSVVRKIENMPTQ-ADKPTTDVTIA
          	*:**. *******:********* ******:*:.******::**: *: .*:*.... *.

MG08104.1 	DCGELPADAVVGDAAKEADKYGDKYEDFPED--DEGGPLDVARVTTIASDCKTYGNSAFK
NCU03853.1	DCGELSGDAAVSADTKTADAYGDEYEDFPEDEATDGQPLSASKILKIATDCKDFGNKAFK
FG10352.1 	DCGELTGDAALAADVKQPDALGDPYEDFPED---CTDTLDAVAVLKIAKASKDYGNTAFK
AN4583.1  	ECGELTGEDYDNADKQTPDATGDPYEDFPDD--HQGEELSAPVCFKIASELKNFGNTAFK
          	:****..:       : .*  ** *****:*       *..    .**.  * :**.***

MG08104.1 	VGDHATALTKYQKGLRYLNEEPERDQENIPADVKEKLAALRFTLNNNAALMNLKLESWED
NCU03853.1	AGDLPVALDKYQKGLRYLNEDPELDNE--PADTKQKLDALRVSLNSNAALMNMKLSAWDE
FG10352.1 	SGNYSLGLDKYQKGLRYINEEPELEDQ--PQSIKDELEALRFSLNNNSALLNIKLEAWDD
AN4583.1  	NGNIALGLEKYQKGLRYLNEFPEPEEND-PKDLEPQMKSLRFTLHSNSSLLANKLGQFKN
          	 *: . .* ********:** ** :::. * . : :: :**.:*:.*::*:  **  :.:

MG08104.1 	AKNAASSALAVK---SASDADRAKALYRRGLAYIRLKDEDAAIKDLEEAKKLAPADAAVA
NCU03853.1	CIRSADGALAVA---TISDKDRAKALYRRGYAQVRIKDEDSALTSLEEAKKLAPEDGAIV
FG10352.1 	AARSASAALEVG---GVKDADRAKAFYRRGLANIHLKDEEAAVRDLTEANKLAPNDSAIT
AN4583.1  	GKTWATYALDVADAASAKDADRAKVYYRRAVAESGLKEEDEALKDLEQASTLAPSDAAIA
          	    *  ** * .::  .* ****. ***. *   :*:*: *: .* :*..*** *.*:.

MG08104.1 	KELEQVKKAAAARAAKEKAAYKKFFS
NCU03853.1	NELAAVKKAAAARMAKEKAAYKKFFS
FG10352.1 	RELNGVKTKAAARAAKEKAAYKKFFT
AN4583.1  	AETARVKKAIKAREAQEKATARKFFS
          	 *   **.   ** *:***: :***:
```
